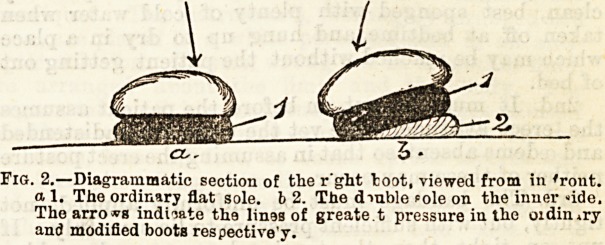# The Treatment of Flat Foot

**Published:** 1892-10-29

**Authors:** 


### THE HOSPITAL CLINIC.

[*The Editor will be glad to receive offers of co-operation and contributions from members of the profession. All letters should be addressed to* The Editor, The Lodge, Porchester Square, London, W.]

### ROYAL INFIRMARY, EDINBURGH.

### The Treatment of Flat Foot.

The treatment of flat or splay foot demands much patience and perseverance from both patient and surgeon. The condition is oftenest met with in message boys or girls between the ages of thirteen and eighteen, young nursery maids, shop girls, and domestic servants. In all these classes the employment necessitates long continued standing or walking, with, in some instances, the carrying of heavy weights. Too rapid growth, recent severe illness, and want of sufficient nourishment are circumstances which exert an important predisposing effect in almost all cases. These unfavourable conditions result in over fatigue and loss of tone in the muscles of the leg, more especially those of the calf, whose tendons, passing towards the sole of the foot, play an important part in maintaining the normal arched condition of the instep. The ligaments of the foot, particularly the inferior calcaneo-scaphoid ligament, become stretched and painful; the position of the bones is so altered that the arch of the foot is gradually obliterated, and ultimately entirely lost. A valuable aid in the diagnosis of this condition is to

Fig. 1.—*a* The impress of a healthy foot. *b* The impress of a "flat foot," showing the absence of the normal hollow on the inner side.

compare the imprint of the affected foot with that of a normal one. This is readily done by smearing ink over the sole, and then causing the patient to stand erect on a sheet of paper. The print of a flat foot wants the well-marked hollow seen along the inner side in a healthy one. A series of such impressions taken at intervals forms an interesting and reliable record of the result of treatment.

The first and most important point to attend to in the treatment of flat foot, is the general hygiene of the patient, using this term in its widest sense, as without this no surgical treatment is of any avail. If the patient cannot afford to be idle, some work must be selected which will admit, if not of constant sitting, at least of periodic rest. Plenty of good nourishing food must be given, augmented, if necessary, by cod-liver oil, maltine, or other strong artificial diet. When the general health is run down, and the appetite deficient, a teaspoonful of Easton's syrup, or a tablespoonful of some such tonic mixture as the following may be given half an hour after each meal: ℞. tinct. Ferri. Perchlor: ʒiii; Tinct. Nucis. Vom: ʒii; Inf. Quassiae ad: ℥vi; mix. An occasional aperient is given if necessary.

Having regulated the general health, the next indication is to remove as much pressure as possible from the defaulting calcaneo-scaphoid ligament. This, of course, is most completely done by proscribing the erect posture entirely, but such a measure is too severe. It will usually be found sufficient to keep the patient as much off his feet as possible, and to direct the weight of his body so that it will not fall on the inner side of the foot. This is done by having the sole of the boot made twice as thick on the inner side as it is on the outer, in other words, a wedge-shaped sole is added to the ordinary boot, the base of the wedge

Fig. 2.—Diagrammatic section of the r'ght boot, viewed from in front. *a* 1. The ordinary flat sole. *b* 2. The double sole on the inner side. The arrows indicate the lines of greatest pressure in the ordinary and modified boots respectively.

being along the inner side. The boots should have very low heels. The effect of this device is to throw the weight of the body on the outer side of the foot, so relieving the ligaments on the inner side.

In addition to all this the muscles of the leg and foot must have their tone improved by regular and systematic exercise so that they may bear their proper share in maintaining the shape of the foot. It is in the conscientious carrying out of this part of the treatment that the secret of success lies; unfortunately, however, it also taxes most the patience of the sufferer. First, the patient, barefooted, standing erect with his feet together, very slowly raises himself on to his tip-toes, and as slowly allows himself to come down again. This movement is repeated 20, 30, or 40 times every night and morning for several weeks. After resting a few minutes he walks up and down the room several times on tip-toe, taking 80, 100, or 120 steps in all. This is done two or three times every day.

The feet and legs are passively put through all the movements possible at the knee and ankle joints, and the muscles regularly massaged. Cold water douches are often found advantageous.

If these three indications—improving the general health, removing pressure from the inner side of the foot, and restoring to tone of the muscles be thoroughly carried out, it is seldom, if ever, necessary, or indeed justifiable, to adopt operative measures.

Various measures may be taken to palliate the condition, for example, the wearing of a strong elastic bandage, applied in the form of a figure-of-8, so as to support the inner side of the foot; a pad of cork, india-rubber, or felt inside the boot, or a steel spring fixed only behind, each of which acts as a buttress to support the falling arch. Of these the last is the best, but all are inferior to the manipulative treatment just described.

Flat foot is often associated with infantile paralysis, knock-knee, and sometimes with spinal curvature, in which case, of course, these various conditions require special treatment.

## Figures and Tables

**Fig. 1. f1:**
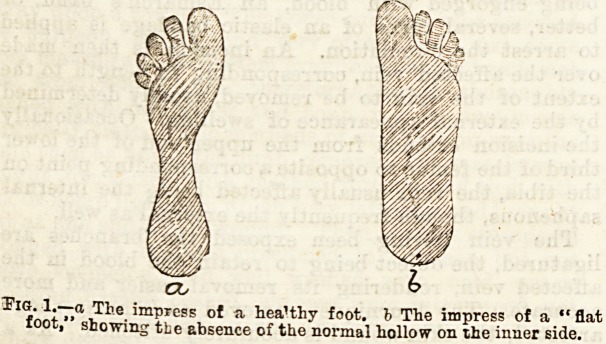


**Fig. 2. f2:**